# Scattering of guided waves propagating through pipe bends based on normal mode expansion

**DOI:** 10.1038/s41598-022-16708-z

**Published:** 2022-07-21

**Authors:** Wenjun Wu, Hao Dong, Shangyu Zhang

**Affiliations:** School of Naval Architecture, Ocean and Energy Power Engineering, Wuhan University of Technology, Wuhan, 430063 China

**Keywords:** Civil engineering, Mechanical engineering

## Abstract

The scattering of guided waves propagating through pipe bends is studied by means of normal mode expansion. First, the bi-orthogonality relationship for normal modes in pipe bends is derived, based on which the displacement and stress fields at the interfaces between the straight and curved parts are expanded with the normal modes in both parts. Then, based on the displacement and stress field continuity principle, the scattering problem is regarded as an eigenproblem of a transfer matrix, the solution of which gives the mode conversions at the interfaces. A case study is presented of the low-frequency longitudinal mode incident on a pipe bend, and it is found that the dominant mode conversions are L(0,1) reflection and mode conversion from L(0,1) to F(1,1). Finite element simulations and experiments are also conducted. L(0,1) bend reflection and mode-converted F(1,1) are clearly observed, which agrees well with the theoretical predictions.

## Introduction

Because it is highly efficient and can detect zones that would otherwise be inaccessible, guided-wave technology^1–3^ is used extensively for inspecting pipelines. However, practical pipelines always have multiple bends that interfere with the propagation of the incident guided wave and thus complicate the testing signals significantly and even make them impossible to interpret. Therefore, the scattering mechanics of guided waves propagating through pipe bends are essential when inspecting complicated pipelines.

Because of the curved axis of a pipe bend, the wave motion therein is much more complex and must be investigated numerically rather than analytically. Demma et al.^4^ first derived the dispersion curves and mode structures of guided waves in pipe bends with the mode analysis method^5^ in commercial finite-element software, but the dispersion relationship can be calculated only at discrete frequencies. Hayashi et al.^6^ first calculated the dispersion curves of guided waves in pipe bends by using the semi-analytic finite-element method (SAFE)^6–10^, which requires only the pipe cross section to be discretized, thereby turning a three-dimensional (3D) problem into a two-dimensional (2D) one and hence saving computational time and memory. A curved cylindrical coordinate system is introduced for the curved pipe region, under which the governing equation of wave motion in pipe bends is derived and then solved with the SAFE method. That method is also applied to dispersion calculations of helical structures^8^ and structures with constant cross sections, such as rails^9^ and square tubes^10^.

Compared with the dispersion curves of guided waves in straight pipes, those for pipe bends exhibit several distinct features, such as cut-off frequencies for the fundamental modes [L(0,1) and T(0,1)], mode splitting^11^, mode repulsion^9^, and natural focusing^12^. Demma et al.^11^ studied the mode-splitting feature and gave the explanation that the originally identical modes in straight pipes split into two different modes because of the loss of axisymmetry in pipe bends. Mode repulsion has also been observed in the dispersion curves for curved plates^13,14^, helical waveguides^8^, and rails^9^, among others. Loveday et al.^9^ studied the mode repulsion of guided waves in rails, following which Wu et al.^15^ studied the same in pipe bends. It is found that mode repulsion occurs when the second derivative of frequency with respect to wavenumber approaches infinity as the two curves approach each other. It is also found that mode repulsion occurs only between modes of the same type (e.g., symmetric or antisymmetric modes) and not between modes of different types (e.g., symmetric and antisymmetric modes).

Although the propagation characteristics of guided waves in pipe bends are well known, the corresponding scattering mechanics remain less understood. Most studies of scattering mechanics are based on numerical simulations^16–20^ and experiments^21–25^. By means of 3D finite-element simulation, Aristegui et al.^16^ simulated the L(0,2) mode traveling across pipe bends and observed mode conversions from L(0,2) to F(1,3) and F(2,3). Demma et al.^11^ studied scattering of the torsional T(0,1) mode and found that it is more likely to be converted to F(1,2). Based on the definition of travel-time-preserving orthogonal parametric representations of curved tubes, Brath et al.^12^ models guided wave propagation and scattering in a bend with a 2-Dimensional approaches. Qi et al.^17^ and Heinlein et al.^18^ investigated reflection of the T(0,1) mode from circumferential and axial defects in pipe bends, respectively. As well as the finite-element method, other numerical methods are also employed: Rudd et al.^19^ used elastodynamic finite integration to simulate guided waves in pipe bends, and Zhou et al.^20^ used the wave finite-element method to study the scattering mechanics of pipe bends.

As for experimental studies, Nishino^21^ used a laser system to generate and sense guided waves in a stainless-steel pipe, and mode conversions in pipe bends were observed. Also using a laser system, Kim et al.^22^ assessed wall-thinning defects in pipe bends. Verma et al.^23^ generated the L(0,2) mode with magnetostrictive transducers and investigated how the bend angle and radii affected the reflection and transmission coefficients. Similarly, Wu et al.^24,25^ used a magnetostrictive system to study scattering of the L(0,1) and T(0,1) modes passing through pipe bends. Brath et al.^26^ experimentally mapped the pipe elbows with a fast forward model-based guided wave tomography method.

Based on a bi-orthogonal relationship, the normal-mode expansion (NME) method expresses the wave motion in a waveguide with orthogonal guided wave modes, thereby facilitating force response analysis. Ditri et al.^27^ first derived the bi-orthogonal relationship in hollow cylinders based on the reciprocity theorem^28^, followed by a generalized mode excitation analysis of pipes with surface traction applied. More specifically, Ditri et al.^29^ analyzed the mode excitation of wedge- and comb-type transducers. Using the same NME method, Zhang et al.^30^ analyzed the force response of elastic hollow cylinders with respect to magnetostrictive loading. Ma et al.^31^ studied the excitation of torsional guided waves in pipes by reversed shear loading. Bakkali et al.^32^ studied the scattering at the junction between straight and curved pipes based on the bi-orthogonal relationship which is simply extended from the bi-orthogonal relationship in plates. Recently, Zhang et al.^33^ used the NME method to study forced guided-wave problems in loading zones and found that the classic NME solution does not satisfy Hooke’s law inside the loading zone. To address this shortcoming, Zhang et al.^34^ proposed a modified NME method.

Herein, the NME method is used to study the scattering mechanics of guided waves in pipe bends. In “SAFE modeling of wave motion in pipe bends” section, the SAFE modeling of wave motion in pipe bends is introduced briefly, then the bi-orthogonal relationship of normal modes in pipe bends is derived in “Bi-orthogonality relationship for normal modes in pipe bends” section. Based on that relationship, a theoretical study of the scattering mechanics is presented in “Theoretical study of guided-wave scattering at pipe bends” section. To illustrate the theoretical scattering study further, a case study of a low-frequency longitudinal mode incident on a small-radius pipe bend is presented in “Case study” section. Finally, in “Numerical simulations” and “Experimental validation” sections, numerical simulations and experiments, respectively, are reported to validate the theoretical predictions.

## SAFE modeling of wave motion in pipe bends

**Figure 1:**
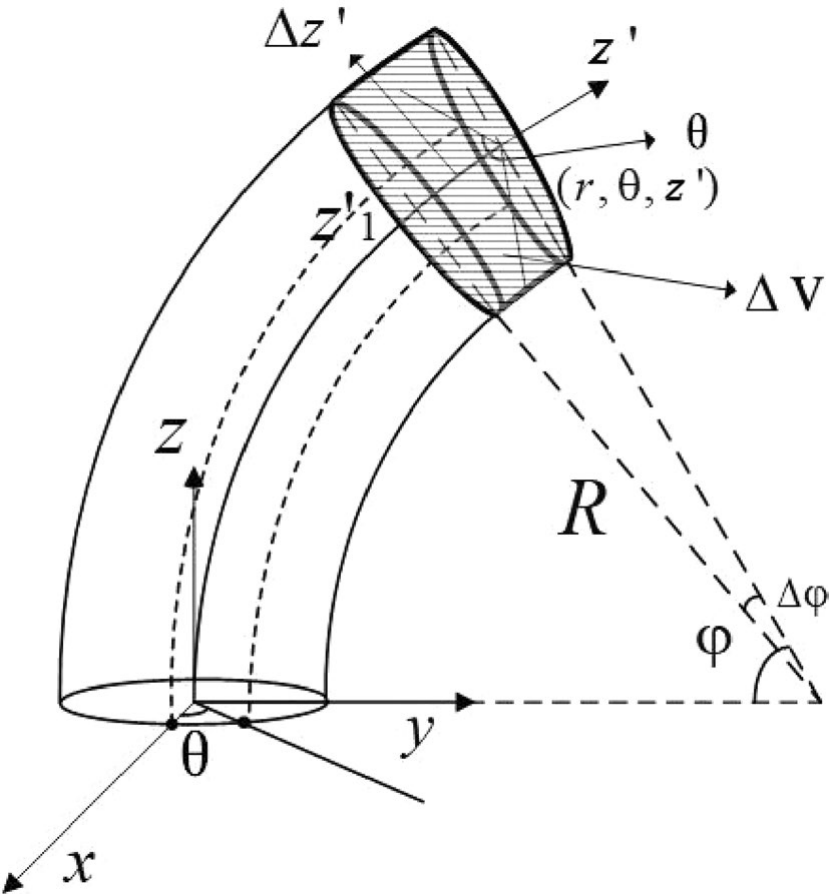
Quasi-cylindrical coordinate system^6^.

As shown in Fig. 1, a quasi-cylindrical coordinate system^6^ is introduced to model the curved hollow cylinder, where the straight *z* axis in cylindrical coordinates is replaced by a curved *z*′ axis along the curvature of the bend. Thus, an arbitrary point (*x*, *y*, *z*) in Cartesian coordinates can be expressed in quasi-cylindrical coordinates (*r*, *θ*,* z*′) as

1 $$\left. \begin{gathered} x = r\cos \theta \hfill \\ y = r\sin \theta \cos \varphi + R\left( {1 - \cos \varphi } \right) \hfill \\ z = - r\sin \theta \sin \varphi + R\sin \theta \hfill \\ R = z^{\prime}/\varphi \hfill \\ \end{gathered} \right\},$$

where *R* is the bending radius.

In quasi-cylindrical coordinates, the stress–displacement relations are rewritten as^6^

2 $${{\varvec{\upsigma}}}{ = }{\mathbf{D}}\left[ {L + L_{r} \frac{\partial }{\partial r} + L_{\theta } \frac{\partial }{\partial \theta } + L_{z^{\prime}} \frac{\partial }{\partial z^{\prime}}} \right]{\mathbf{u}},$$

where ${\mathbf{u}} = \left[ {u_{r} ,u_{\theta } ,u_{z^{\prime}} } \right]^{T}$ is the displacement vector, ${{\varvec{\upsigma}}} = \left[ {\sigma_{rr} ,\sigma_{\theta \theta } ,\sigma_{\theta z^{\prime}} ,\sigma_{\theta z^{\prime}} ,\sigma_{z^{\prime}r} ,\sigma_{r\theta } } \right]^{T}$ is the stress vector,

$$L = \left[ {\begin{array}{*{20}c} 0 & 0 & 0 \\ \frac{1}{r} & 0 & 0 \\ { - \frac{\sin \theta }{{Rh}}} & { - \frac{\cos \theta }{{Rh}}} & 0 \\ 0 & 0 & {\frac{\cos \theta }{{Rh}}} \\ 0 & 0 & {\frac{\sin \theta }{{Rh}}} \\ 0 & { - \frac{1}{r}} & 0 \\ \end{array} } \right],\quad L_{r} = \left[ {\begin{array}{*{20}c} 1 & 0 & 0 \\ 0 & 0 & 0 \\ 0 & 0 & 0 \\ 0 & 0 & 0 \\ 0 & 0 & 1 \\ 0 & 1 & 0 \\ \end{array} } \right],$$

3 $$L_{\theta } = \left[ {\begin{array}{*{20}c} {0} & 0 & 0 \\ 0 & {\frac{{1}}{r}} & 0 \\ 0 & 0 & 0 \\ 0 & 0 & \frac{1}{r} \\ 0 & 0 & 0 \\ \frac{1}{r} & 0 & 0 \\ \end{array} } \right],\quad L_{z^{\prime}} = \left[ {\begin{array}{*{20}c} {0} & 0 & 0 \\ 0 & 0 & 0 \\ 0 & 0 & \frac{1}{h} \\ 0 & \frac{1}{h} & 0 \\ \frac{1}{h} & 0 & 0 \\ 0 & 0 & 0 \\ \end{array} } \right],\,{\text{and}}\,h = 1 - \frac{r\sin \theta }{R},$$

${\mathbf{D}}$ is the constitutive equation, which is defined as

$${\mathbf{D}} = \left[ {\begin{array}{*{20}c} {\lambda + 2\mu } & \lambda & \lambda & 0 & 0 & 0 \\ \lambda & {\lambda + 2\mu } & \lambda & 0 & 0 & 0 \\ \lambda & \lambda & {\lambda + 2\mu } & 0 & 0 & 0 \\ 0 & 0 & 0 & \mu & 0 & 0 \\ 0 & 0 & 0 & 0 & \mu & 0 \\ 0 & 0 & 0 & 0 & 0 & \mu \\ \end{array} } \right],$$

where *λ* is the Poisson ratio and *µ* is the shear modulus of elasticity.

Guided waves are assumed to propagate along the curved axis, hence the displacement **u** in a pipe bend takes the form

4 $${\mathbf{u}} = {\mathbf{U}}\left( {r,\theta } \right)\exp (ikz^{\prime})\exp ( - i\omega t),$$

where *k* is the wavenumber, $\omega$ is the angular frequency, and ${\mathbf{U}}\left( {r,\theta } \right)$ is the interpolated displacement in the cross section of the waveguide. Because the wave motion in the *z*′ direction is assumed to be harmonic, finite-element discretization is required only over the cross section of the pipe bend, with the harmonic wave motion in the *z*′ direction included analytically. Because only the cross section is discretized and not a volume, thereby turning a 3D problem into a 2D one, this method decreases the number of nodes significantly and hence saves computational time and memory.

With the rewritten strain–displacement relation [Eq. (2)] and following the standard procedure of the finite-element method, the governing equation of wave motion in pipe bends can be written as^4^

5 $$\left\{ {{\mathbf{K}}_{1}^{{}} + ik{\mathbf{K}}_{2}^{{}} + k^{2} {\mathbf{K}}_{3}^{{}} - \omega^{2} {\mathbf{M}}^{{}} } \right\}{\mathbf{U}}{ = 0,}$$

where **U** is the nodal displacement, **K**_1_, **K**_2_, and **K**_3_ are the stiffness matrices, and **M** is the mass matrix. The stiffness and mass matrices are all real and symmetric, except that **K**_2_ is antisymmetric. Let ${\mathbf{K}}_{{2}}^{^{\prime}} = i{\mathbf{K}}_{{2}}^{{}}$, then ${\mathbf{K}}_{{2}}^{^{\prime}}$ is conjugate symmetric. Thus, all the matrices in Eq. (5) can be considered as being conjugate symmetric. The governing equation of Eq. (5) can be regarded as an eigenvalue problem and can be rewritten as

6 $$\left( {{\mathbf{K}}_{1}^{{}} + k{\mathbf{K}}_{2}^{^{\prime}} + k^{2} {\mathbf{K}}_{3}^{{}} } \right){{\varvec{\uppsi}}} = \lambda {\mathbf{M\psi }},$$

where $\lambda = \omega^{2}$ is the eigenvalue and ${{\varvec{\uppsi}}}$ is the eigenvector, which also represents the mode structure. Then the wavenumber–frequency dispersion curves and the corresponding mode structure can be computed by solving this eigenvalue problem.

## Bi-orthogonality relationship for normal modes in pipe bends

The NME method is based on a bi-orthogonal relationship and expresses the wave motion with orthogonal guided wave modes. The bi-orthogonality relationship for normal modes in pipe bends is derived in this section by following the work of Ref.^27^, in which the bi-orthogonality relationship for a straight pipe is deduced.

### Bi-orthogonality relationship for pipe bends

The bi-orthogonality relationship for normal modes is derived from the complex reciprocity relation^27^, which states that

7 $$\nabla \cdot \left( {{\mathbf{v}}_{2}^{*} \cdot {{\varvec{\upsigma}}}_{1} + {\mathbf{v}}_{1} \cdot {{\varvec{\upsigma}}}_{2}^{*} } \right) = 0,$$

where **V**_1_, ${{\varvec{\upsigma}}}_{1}$ and **V**_2_, ${{\varvec{\upsigma}}}_{2}$ are the particle velocity and stress fields, respectively, of two different wave motions in a linearly elastic waveguide, and the asterisk represents complex conjugation.

Let ${\mathbf{V}}_{1}$, ${{\varvec{\upsigma}}}_{1}$ and ${\mathbf{V}}_{2}$, ${{\varvec{\upsigma}}}_{2}$ be different modes in a pipe bend. In quasi-cylindrical coordinates, they take the forms of

8 $$\begin{gathered} {\mathbf{v}}_{1} = {\mathbf{V}}_{1} e^{{ - ik_{1} z^{\prime}}} ,\quad {{\varvec{\upsigma}}}_{1} = {\mathbf{T}}_{1} e^{{ - ik_{1} z^{\prime}}} , \hfill \\ {\mathbf{v}}_{{2}} = {\mathbf{V}}_{2} e^{{ - ik_{2} z^{\prime}}} ,\quad {{\varvec{\upsigma}}}_{{2}} = {\mathbf{T}}_{2} e^{{ - ik_{2} z^{\prime}}} , \hfill \\ \end{gathered}$$

where *k* is the modal wavenumber, and **V** and **T** are the particle velocity and stress fields, respectively, over the cross section of the pipe bend. Note that here and hereinafter, the harmonic time dependence $e^{ - iwt}$ is omitted for brevity.

Combining Eqs. (7) and (8) gives

9 $$\nabla \cdot \left( {{\mathbf{v}}_{2}^{*} \cdot {{\varvec{\upsigma}}}_{1} + {\mathbf{v}}_{1} \cdot {{\varvec{\upsigma}}}_{2}^{*} } \right) = \nabla \cdot \left\{ {e^{{ - i(k_{1} - k_{2}^{*} )z^{\prime}}} \left( {{\mathbf{V}}_{2}^{*} \cdot {\mathbf{T}}_{1} + {\mathbf{V}}_{1} \cdot {\mathbf{T}}_{2}^{*} } \right)} \right\} = 0.$$

Integrating Eq. (9) over a slice in the pipe bend (the Δ*V* volume in Fig. 1) gives

10 $$\iiint\limits_{\Delta V} {\nabla \cdot \left\{ {e^{{ - i(k_{1} - k_{2}^{*} )z^{\prime}}} \left( {{\mathbf{V}}_{2}^{*} \cdot {\mathbf{T}}_{1} + {\mathbf{V}}_{1} \cdot {\mathbf{T}}_{2}^{*} } \right)} \right\}dV} = 0.$$

By using Gauss’s divergence theorem, the volume integral in the Δ*V* volume becomes the area integral over its surface, i.e.,

11 $$\begin{aligned} \iiint\limits_{\Delta V} {\nabla \cdot \left\{ {e^{{ - i(k_{1} - k_{2}^{*} )z^{\prime}}} \left( {{\mathbf{V}}_{2}^{*} \cdot {\mathbf{T}}_{1} + {\mathbf{V}}_{1} \cdot {\mathbf{T}}_{2}^{*} } \right)} \right\}}dV & = e^{{ - i(k_{1} - k_{2}^{*} )z^{\prime}}} \iint\limits_{{\partial S_{1} + \partial S_{2} }} {\left( {{\mathbf{V}}_{2}^{*} \cdot {\mathbf{T}}_{1} + {\mathbf{V}}_{1} \cdot {\mathbf{T}}_{2}^{*} } \right) \cdot \hat{n}ds} \\ & \quad + \,\left( \begin{gathered} \iint\limits_{{\partial S_{{z^{\prime}_{1} + \Delta z^{\prime}}} }} {e^{{ - i(k_{1} - k_{2}^{*} )z^{\prime}}} \left( {{\mathbf{V}}_{2}^{*} \cdot {\mathbf{T}}_{1} + {\mathbf{V}}_{1} \cdot {\mathbf{T}}_{2}^{*} } \right) \cdot \hat{e}_{z^{\prime}} ds} \hfill \\ - \iint\limits_{{\partial S_{{z^{\prime}_{1} }} }} {e^{{ - i(k_{1} - k_{2}^{*} )z^{\prime}}} \left( {{\mathbf{V}}_{2}^{*} \cdot {\mathbf{T}}_{1} + {\mathbf{V}}_{1} \cdot {\mathbf{T}}_{2}^{*} } \right) \cdot \hat{e}_{z^{\prime}} ds} \hfill \\ \end{gathered} \right), \\ \end{aligned}$$

where $\partial S_{1}$ and $\partial S_{2}$ are the outer and inner surfaces, respectively, of the Δ*V* volume, $\partial S_{{z^{\prime}_{1} }}$ and $\partial S_{{z^{\prime}_{1} + \Delta z^{\prime}}}$ are the cross sections of the pipe bend at the *z*′ positions of $z^{\prime}_{1}$ and $z^{\prime}_{1} + \Delta z^{\prime}$, respectively, $\hat{e}_{z^{\prime}}$ is the unit vector in the *z*′ direction, and $\hat{n}$ is the unit normal vector pointing away from the interior volume.

For a free pipe bend, its inner and outer surfaces experience no traction, therefore the first term on the right-hand side of Eq. (11) vanishes. Moreover, because the term ${\mathbf{V}}_{2}^{*} \cdot {\mathbf{T}}_{1} + {\mathbf{V}}_{1} \cdot {\mathbf{T}}_{2}^{*}$ is independent of *z*′, its area integrals in $\partial S_{{z^{\prime}_{1} }}$ and $\partial S_{{z^{\prime}_{1} + \Delta z^{\prime}}}$ are the same. Therefore, Eq. (11) can be written as

12 $$\left( {e^{{ - i(k_{1} - k_{2}^{*} )z^{\prime}_{1} }} - e^{{ - i(k_{1} - k_{2}^{*} )\left( {z^{\prime}_{1} + \Delta z^{\prime}} \right)}} } \right)\iint\limits_{{\partial S_{{z^{\prime}_{1} }} }} {\left( {{\mathbf{V}}_{2}^{*} \cdot {\mathbf{T}}_{1} + {\mathbf{V}}_{1} \cdot {\mathbf{T}}_{2}^{*} } \right) \cdot \hat{e}_{z^{\prime}} ds} = 0.$$

Letting $\Delta z^{\prime} \to 0$, Eq. (12) still holds and becomes

13 $$- i\left( {k_{1} - k_{2}^{*} } \right){\mathbf{P}}_{{k_{1} ,k_{2} }} = 0,$$

where

14 $${\mathbf{P}}_{{k_{1} ,k_{2} }} { = }\iint_{s} {\left( {{\mathbf{V}}_{2}^{*} \cdot {\mathbf{T}}_{1} + {\mathbf{V}}_{1} \cdot {\mathbf{T}}_{2}^{*} } \right)} \cdot \hat{e}_{z^{\prime}} ds.$$

Equation (14) indicates that

15 $${\mathbf{P}}_{{k_{1} ,k_{2} }} = {0}\,\,{\text{unless}}\,\,k_{1}^{{}} = k_{2}^{ * } .$$

Equation (15) is the bi-orthogonality relationship for normal modes in pipe bends.

### Numerical validation for the bi-orthogonality relationship

In this subsection, the bi-orthogonality relationship of Eq. (15) is validated numerically by investigating a stainless-steel pipe with an outer diameter of 22 mm, a thickness of 2 mm, and a bend radius of 50 mm. The material properties of the stainless-steel pipe are given in Table 1.

**Table 1:** Material properties of stainless-steel pipe.

Young’s modulus	Poisson’s ratio	Density
206 GPa	0.27	7930 kg/m^3^

**Figure 2:**
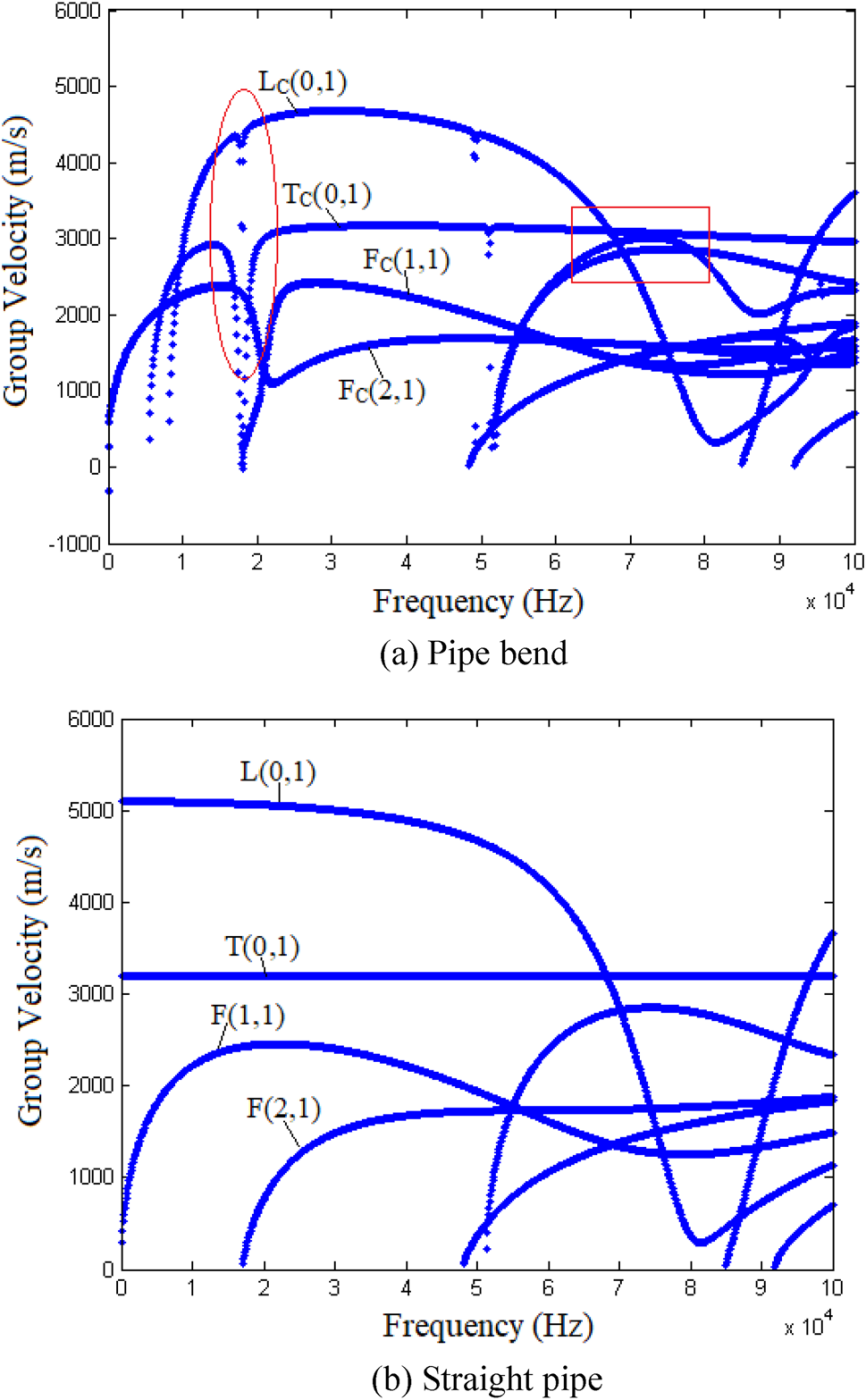
Group-velocity dispersion curves for (**a**) a pipe bend and (**b**) a straight pipe.

The wave motion in the pipe bend is derived using the SAFE method introduced in “SAFE modeling of wave motion in pipe bends” section. The SAFE method is implemented with Matlab codes. The cross section of the pipe bend is first discretized with two elements in the radial direction and 48 elements in the circumferential direction. By solving the eigenvalue problem [Eq. (6)], the dispersion relationship for the pipe bend is derived. The group-velocity dispersion curves for the pipe bend are shown in Fig. 2a, and those for the straight pipe are shown in Fig. 2b. For comparison, the modes in the pipe bend are denoted as those in the straight pipe but with the addition of the subscript C, as shown in Fig. 2a. As are clear in Fig. 2a, the distinct characteristics of the dispersion curves for the pipe bend are (i) the cut-off frequencies evident for the T_C_(0,1) and L_C_(0,1) modes, (ii) the mode-splitting phenomena marked with the frames, and (iii) the mode-repulsion phenomena marked with the circles. Note that Fig. 2a shows only the positive propagating modes, but all the modes including the negative propagating and non-propagating ones are investigated in the validation of the bi-orthogonality relationship.

**Figure 3:**
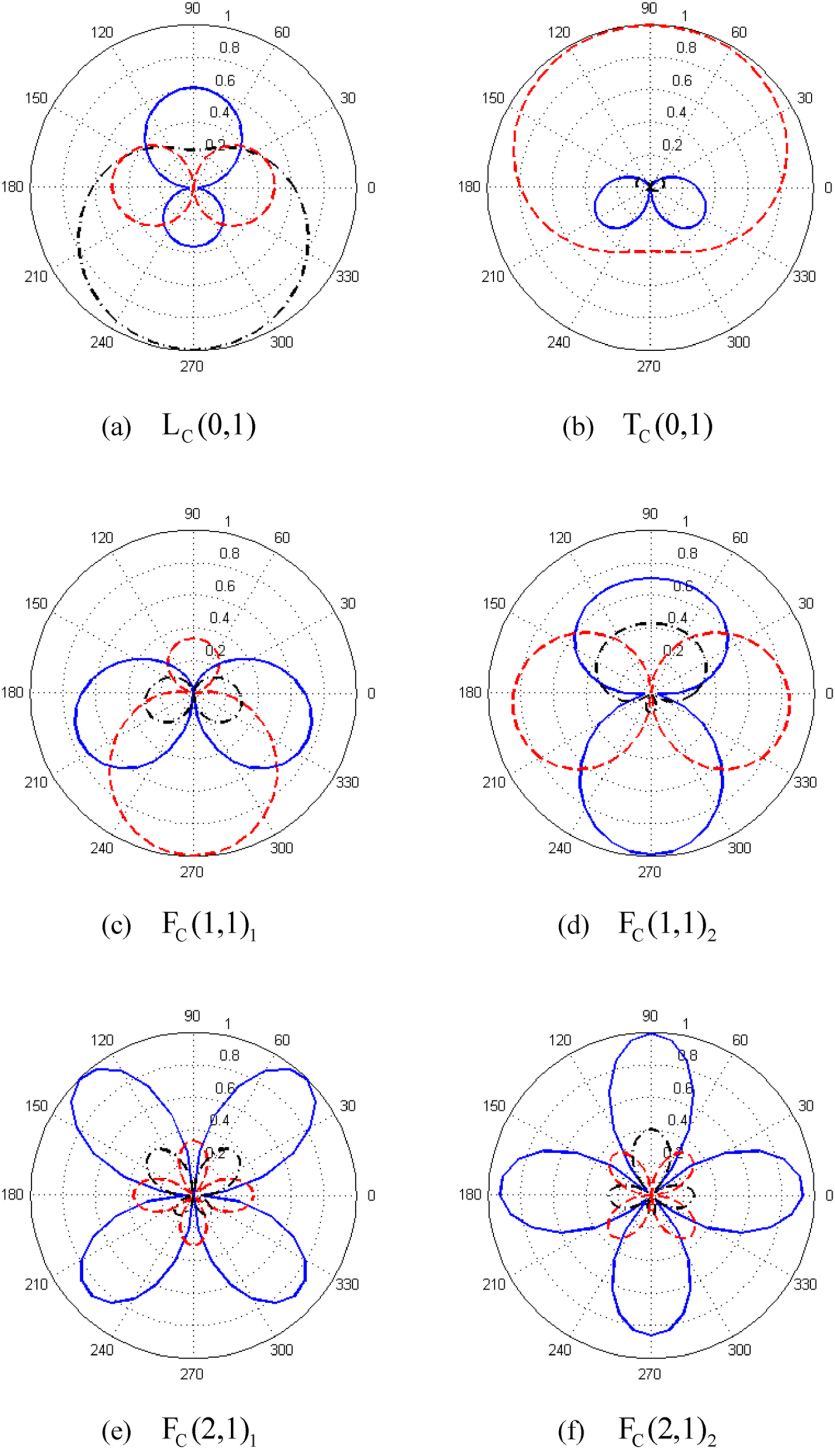
Displacement distributions along circumferential direction (the blue solid, red dotted, and black dashed-dotted lines show the displacements in the radial, circumferential, and axial directions, respectively).

The excitation frequency of 30 kHz is chosen to investigate the bi-orthogonality relationship. Also, by solving Eq. (6), the mode structure (eigenvector ${{\varvec{\uppsi}}}$) is deduced. Figure 3 shows the displacement distribution along the circumferential direction for the (a) ${\text{L}}_{{\text{C}}} {(0,1)}$, (b) ${\text{T}}_{{\text{C}}} {(0,1)}$, (c) ${\text{F}}_{{\text{C}}} {(1,1)}_{1}$, (d) ${\text{F}}_{{\text{C}}} {(1,1)}_{2}$, (e) ${\text{F}}_{{\text{C}}} {(2,1)}_{1}$, and (f) ${\text{F}}_{{\text{C}}} {(2,1)}_{2}$ modes.

The term ${\mathbf{P}}_{{k_{1} ,k_{2} }}$ [Eq. (14)] in the bi-orthogonality relationship is an integral over the cross section, which can be calculated by following the SAFE calculation procedure. For each element on the cross section, we have

16 $$\begin{aligned} {\mathbf{P}}_{{k_{1} ,k_{2} }}^{e} & { = }\iint_{{s^{e} }} {\left( {{\mathbf{V}}_{2}^{e,*} \cdot {\mathbf{T}}_{1}^{e} + {\mathbf{V}}_{1}^{e} \cdot {\mathbf{T}}_{2}^{e,*} } \right)} \cdot \hat{e}_{z^{\prime}} ds^{e} \\ & = j\omega \cdot \iint_{{s^{e} }} {\left\{ \begin{gathered} {{\varvec{\uppsi}}}_{2}^{e,*} \cdot \left[ {{\mathbf{D}}\left( {L + L_{r} \frac{\partial }{\partial r} + L_{\theta } \frac{\partial }{\partial \theta } + ik_{m}^{N} L_{z^{\prime}} } \right){{\varvec{\uppsi}}}_{1}^{e} } \right] \hfill \\ + {{\varvec{\uppsi}}}_{1}^{e} \cdot \left[ {{\mathbf{D}}\left( {L + L_{r} \frac{\partial }{\partial r} + L_{\theta } \frac{\partial }{\partial \theta } + ik_{m}^{N} L_{z^{\prime}} } \right){{\varvec{\uppsi}}}_{2}^{e} } \right]^{*} \hfill \\ \end{gathered} \right\}} \cdot \hat{e}_{z^{\prime}} ds^{e} , \\ \end{aligned}$$

where the superscript *e* denotes the element and ${{\varvec{\uppsi}}}_{{}}^{e}$ is the displacement vector of the nodes in that element. The integral in Eq. (16) can be calculated numerically as a Gaussian integral. Then, by summing the integrals of all the elements, ${\mathbf{P}}_{{k_{1} ,k_{2} }}$ is obtained.

The ${\mathbf{P}}_{{k_{1} ,k_{2} }}$ values for the normal modes in the pipe bend are calculated with the normalized mode structures ${\overline{\mathbf{\psi }}}_{k}$, which is defined as follows:

17 $$\begin{aligned} & {\overline{\mathbf{\psi }}}_{k} = \frac{{{{\varvec{\uppsi}}}_{k} }}{{{\mathbf{P}}_{k,k} }}\quad {\text{for}}\,{\text{propagating}}\,{\text{modes}}; \\ & {\overline{\mathbf{\psi }}}_{k} = \frac{{{{\varvec{\uppsi}}}_{k} }}{{{\mathbf{P}}_{{k,k^{*} }} }}\quad {\text{for}}\,{\text{non - propagating}}\,{\text{modes}}. \\ \end{aligned}$$

${\mathbf{P}}_{k,k}$ is actually double of the Poynting vector, which is defined as ${\mathbf{P}}{ = }\iint_{s} {{\text{Real}}\left( {{\mathbf{V}}_{{}}^{*} \cdot {\mathbf{T}}} \right)} \cdot \hat{e}_{z^{\prime}} ds$ and denotes the average power over the cross-section. Thus, this normalization is a classical normalization process performed with respect to the square root of the Poynting vector. The ${\mathbf{P}}_{{k_{1} ,k_{2} }}$ values for the different modes are zero, thereby validating the bi-orthogonality relationship.

## Theoretical study of guided-wave scattering at pipe bends

With the bi-orthogonality relationship for pipe bends derived in last section, the incident mode and all possible reflected modes can be expanded with the normal modes in pipe bends at the interfaces. Vise verse, the transmitted modes can be expanded with the normal modes in straight pipes. By taking the displacement and stress continuity principle into account, a transfer matrix between scattering coefficients can be established. Then, by solving the transfer matrix, the mode conversions at the interfaces are deduced.

**Figure 4:**
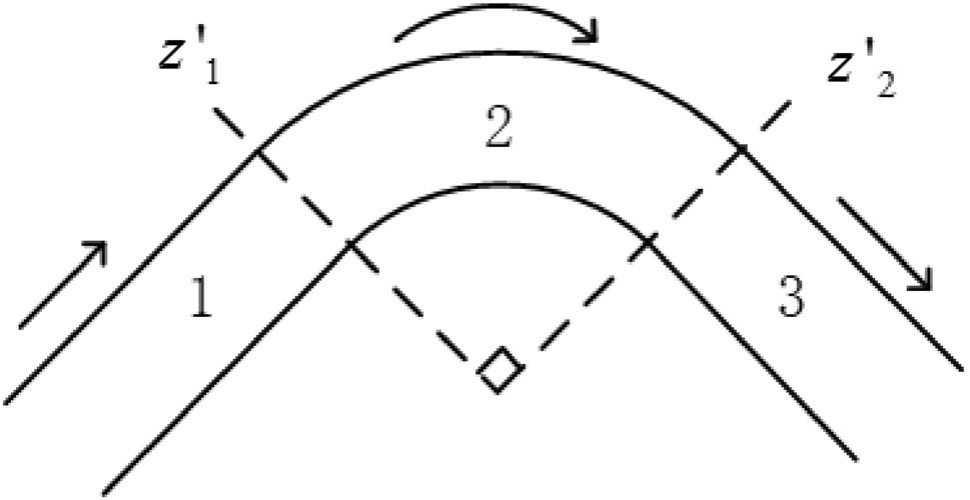
Schematic of guided wave traveling through a bend.

Assume that a guided wave excited in the straight part of a pipe propagates through a pipe bend, as shown in Fig. 4. For one pipe bend, there are two interfaces on the propagating path, as marked by $z^{\prime}_{1}$ and $z^{\prime}_{2}$ in Fig. 4. Complicated mode conversions occur at these interfaces, scattering different modes of the guided wave and causing significant confusion with the testing signals.

### Basic principles for theoretical derivation

For each interface, the displacement and stress fields over the cross section should be consistent, i.e.,

18 $$\left. {{\mathbf{V}}_{i} } \right|_{{z^{\prime} = z^{\prime}_{i} }} = \left. {{\mathbf{V}}_{i + 1} } \right|_{{z^{\prime} = z^{\prime}_{i} }} ,\quad \left. {{\mathbf{T}}_{i} } \right|_{{z^{\prime} = z^{\prime}_{i} }} = \left. {{\mathbf{T}}_{i + 1} } \right|_{{z^{\prime} = z^{\prime}_{i} }} ,\quad (i = 1,2),$$

where the subscript *i* denotes the *i*th section of the pipe shown in Fig. 4. According to the NME method, the mode structures at the interfaces can also be expanded with normal modes in either part, i.e.,

19 $$\left. {\mathbf{V}} \right|_{{z^{\prime} = z^{\prime}_{i} }} = \sum\limits_{n} {\left( {a_{s,n} {\overline{\mathbf{\psi }}}_{{k_{s,n} }} } \right) = } \sum\limits_{n} {\left( {a_{c,n} {\overline{\mathbf{\psi }}}_{{k_{c,n} }} } \right)} ,$$

20 $$\left. {\mathbf{T}} \right|_{{z^{\prime} = z^{\prime}_{i} }} = \sum\limits_{n} {\left( {b_{s,n} {\overline{\mathbf{T}}}_{{k_{s,n} }} } \right) = } \sum\limits_{n} {\left( {b_{c,n} {\overline{\mathbf{T}}}_{{k_{c,n} }} } \right)} ,$$

where *s* and *c* denote the straight pipe and curved pipe, respectively, *a* and *b* are the expansion coefficients of the normal modes, and ${\overline{\mathbf{T}}}$ is the normalized stress mode structure, which in the SAFE modeling is defined as

21 $${\overline{\mathbf{T}}} = {\mathbf{D}}\left[ {L + L_{r} \frac{\partial }{\partial r} + L_{\theta } \frac{\partial }{\partial \theta } + L_{z^{\prime}} \frac{\partial }{\partial z^{\prime}}} \right]{\overline{\mathbf{\psi }}}.$$

Because the displacement–stress relationship [Eq. (2) or (21)] is nonlinear, the expansion coefficients of the displacement mode structures (*a*_*n*_) are different from those of the stress mode structures (*b*_*n*_). However, *b*_*n*_ can be calculated according to the displacement–stress relationship.

Based on the bi-orthogonality relationships of both straight pipes^27^ and pipe bends, the expansion coefficients are calculated as

22 $$a_{s,n} = \iint_{s} {\left( {\left. {{\mathbf{V}}^{*} } \right|_{{z^{\prime} = z^{\prime}_{i} }} \cdot {\overline{\mathbf{T}}}_{{k_{s,n} }} + j\omega \cdot {\overline{\mathbf{\psi }}}_{{k_{s,n} }} \cdot \left. {{\mathbf{T}}^{*} } \right|_{{z^{\prime} = z^{\prime}_{i} }} } \right)} \cdot \hat{e}_{z^{\prime}} ds,$$

23 $$a_{c,n} = \iint_{s} {\left( {\left. {{\mathbf{V}}^{*} } \right|_{{z^{\prime} = z^{\prime}_{i} }} \cdot {\overline{\mathbf{T}}}_{{k_{c,n} }} + j\omega \cdot {\overline{\mathbf{\psi }}}_{{k_{c,n} }} \cdot \left. {{\mathbf{T}}^{*} } \right|_{{z^{\prime} = z^{\prime}_{i} }} } \right)} \cdot \hat{e}_{z^{\prime}} ds.$$

Note that because the normal modes are essentially the solutions to the governing equation of wave motions in waveguides, the normal modes cannot satisfy two different governing equations for different waveguides simultaneously. Assume the displacement field of the interface is expressed by normal modes in straight pipes and pipe bends simultaneously. Then, the stress field of the interface can be expressed by normal modes either in straight pipes or pipe bends. Because the stress field is calculated according to different Hook Laws (different L operators in Eq. (2)), the stress field continuity cannot be ensured. This is to say, the displacement and stress field continuity principle does not hold in the NME framework. However, the NME method still reveals the inherent connections between the modes in straight pipes and pipe bends, and it gives valuable information about the mode conversions at pipe bends. Therefore, the displacement and stress field continuity principle is assumed to hold in the following derivation.

### Scattering study for first interface

At interface $z^{\prime}_{1}$, each mode in straight section 1 can also be expanded with normal modes in curved section 2, i.e.,

24 $${\overline{\mathbf{\psi }}}_{{k_{s,l} }} = \sum\limits_{m} {\left( {a_{lm} {\overline{\mathbf{\psi }}}_{{k_{c,m} }} } \right)} ,$$

25 $$a_{lm} = \iint_{s} {\left( {{\overline{\mathbf{\psi }}}^{*}_{{k_{s,l} }} \cdot {\overline{\mathbf{T}}}_{{k_{c,m} }} + j\omega \cdot {\overline{\mathbf{\psi }}}_{{k_{c,m} }} \cdot {\overline{\mathbf{T}}}^{*}_{{k_{s,l} }} } \right)} \cdot \hat{e}_{z^{\prime}} ds.$$

Then, by combining Eqs. (19) and (24) and considering the continuity principle of Eq. (18), we obtain

26 $$\begin{aligned} \left. {\mathbf{V}} \right|_{{z^{\prime} = z^{\prime}_{1} }} & = \sum\limits_{l} {\left( {a_{s,l} {\overline{\mathbf{\psi }}}_{{k_{s,l} }} } \right) = } \sum\limits_{l} {\left( {a_{s,l} \sum\limits_{m} {\left( {a_{lm} {\overline{\mathbf{\psi }}}_{{k_{c,m} }} } \right)} } \right)} \\ & = \sum\limits_{m} {\left( {\sum\limits_{l} {\left( {a_{s,l} a_{lm} } \right){\overline{\mathbf{\psi }}}_{{k_{c,m} }} } } \right)} = \sum\limits_{m} {\left( {a_{c,m} {\overline{\mathbf{\psi }}}_{{k_{c,m} }} } \right)} . \\ \end{aligned}$$

Equation (26) gives the relationship between the expansion coefficients as

27 $$\sum\limits_{l} {\left( {a_{s,l} {\overline{\mathbf{\psi }}}_{{k_{s,l} }} } \right) = } \sum\limits_{m} {\left( {\sum\limits_{l} {\left( {a_{s,l} a_{lm} } \right){\overline{\mathbf{\psi }}}_{{k_{c,m} }} } } \right)} = \sum\limits_{m} {\left( {a_{c,m} {\overline{\mathbf{\psi }}}_{{k_{c,m} }} } \right)} ,$$

which can be expressed in matrix form as

28 $${\mathbf{A}}_{m} = {\mathbf{A}}_{l} {\mathbf{G}}_{lm} ,$$

where ${\mathbf{A}}_{m} = \left( {a_{c,1}^{t} ,a_{c,2}^{t} , \cdots } \right)$, ${\mathbf{A}}_{l} = \left( {a_{s,1}^{i} ,a_{s,2}^{i} , \cdots ,a_{s,1}^{r} ,a_{s,2}^{r} , \cdots } \right)$, and ${\mathbf{G}}_{lm}$ is the transfer matrix defined as

29 $${\mathbf{G}}_{lm} = \left[ {\begin{array}{*{20}c} {a_{11} } & {a_{12} } & \cdots & {a_{1m} } & \cdots \\ {a_{21} } & {a_{22} } & \cdots & {a_{2m} } & \cdots \\ \vdots & \vdots & \ddots & \vdots & \vdots \\ {a_{l1} } & {a_{l2} } & \cdots & {a_{lm} } & \cdots \\ \vdots & \vdots & \vdots & \vdots & \vdots \\ \end{array} } \right].$$

All modes including the incident positive propagating modes, transmitting positive propagating modes, reflecting negative propagating modes, and non-propagating modes should be considered in the calculation. Therefore, the superscripts *i*, *r*, and *t* are introduced to denote the incident, reflecting, and transmitting modes, respectively.

Conversely, by expanding each mode in pipe section 2 with normal modes in pipe section 1 and following the same derivation procedure, we have

30 $${\mathbf{A}}_{l} = {\mathbf{A}}_{m} {\mathbf{G}}_{lm} = {\mathbf{A}}_{m} {\mathbf{G}}_{lm} ^{\prime}.$$

Combining Eqs. (29) and (30) gives

31 $${\mathbf{A}}_{l} = {\mathbf{A}}_{m} {\mathbf{G}}_{lm} ^{\prime} = {\mathbf{A}}_{l} {\mathbf{G}}_{lm} {\mathbf{G}}_{lm} ^{\prime},$$

which implies that ${\mathbf{A}}_{l}$ is the eigenvector of ${\mathbf{G}}_{lm} {\mathbf{G}}_{lm} ^{\prime}$ with respect to the eigenvalue of one. Thus, by solving the eigenproblem of ${\mathbf{G}}_{lm} {\mathbf{G}}_{lm} ^{\prime}$, the expansion coefficients ${\mathbf{A}}_{l}$ of guided waves in pipe section 1 can be derived, and ${\mathbf{A}}_{m}$ can be calculated according to Eq. (28).

In practical inspections, usually a single mode is excited in pipe section 1. Then, by setting $a_{s,1}^{i}$ in ${\mathbf{A}}_{l}$ to be one and calculating ${\mathbf{A}}_{l}$ and ${\mathbf{A}}_{m}$, the reflecting and transmitting coefficients of guided waves propagating across the first interface are derived.

Considered the acoustic field in either the straight or curved section as being linear, multimode incidence can be treated as multiple single-mode incidences, which can be done by calculating the scattering of each single-mode incidence separately and then linearly superposing these scattering acoustic fields.

### Scattering study for second interface

Because multiple modes are scattered at the first interface, multimode incidence should be considered for the second interface. As mentioned before, multimode incidence is considered as multiple single-mode incidences. For each incident mode *j*, we have

32 $${\mathbf{A}}_{n,j} = {\mathbf{A}}_{m,j} {\mathbf{G}}_{mn,j} ,$$

33 $${\mathbf{A}}_{m,j} = {\mathbf{A}}_{m,j} {\mathbf{G}}_{mn,j} {\mathbf{G}}_{mn,j} ^{\prime},$$

where ${\mathbf{A}}_{n,j} = \left( {a_{s,1}^{t,j} ,a_{s,2}^{t,j} , \cdots } \right)$ are the expansion coefficients of normal modes in straight section 3, and ${\mathbf{A}}_{m,j} = \left( {a_{c}^{i,j} ,a_{c,1}^{r,j} ,a_{c,2}^{r,j} , \cdots } \right)$ are those in curved section 2. Also, by solving the eigenproblem of ${\mathbf{G}}_{mn,j} {\mathbf{G}}_{mn,j} ^{\prime}$, the transmission coefficients ${\mathbf{A}}_{n,j}$ and reflection coefficients $\left( {a_{c,1}^{r,j} ,a_{c,2}^{r,j} , \cdots } \right)$ of the *j*th incident mode scattering at the $z^{\prime}_{2}$ interface are deduced.

By superposing all the scattering acoustic fields, the scattering at the $z^{\prime}_{2}$ interface is obtained. The reflection and transmission coefficients are

34 $${\mathbf{A}}_{n} = \sum\limits_{j} {a_{c}^{i,j} {\mathbf{A}}_{n,j} = } \sum\limits_{j} {a_{c}^{i,j} \left( {a_{s,1}^{t,j} ,a_{s,2}^{t,j} , \cdots } \right) = } \left( {a_{s,1}^{t} ,a_{s,2}^{t} , \cdots } \right),$$

35 $${\mathbf{A}}_{m} = \sum\limits_{j} {a_{c}^{i,j} {\mathbf{A}}_{m,j} = } \sum\limits_{j} {a_{c}^{i,j} \left( {1,a_{s,1}^{t,j} ,a_{s,2}^{t,j} , \cdots } \right) = } \left( {a_{c}^{i,1} ,a_{c}^{i,2} , \cdots a_{s,1}^{t} ,a_{s,2}^{t} , \cdots } \right).$$

The modes reflected at the $z^{\prime}_{2}$ interface then incident negatively on the $z^{\prime}_{1}$ interface, thereby enforcing the reflections of the latter. Because the reflections between the $z^{\prime}_{1}$ and $z^{\prime}_{2}$ interfaces are rather small in most cases, they are neglected for simplification.

Combining the scattering fields of the $z^{\prime}_{1}$ and $z^{\prime}_{2}$ interfaces gives the reflection and transmission coefficients (${\mathbf{A}}_{l}$ and ${\mathbf{A}}_{n}$) of guided waves traveling through the pipe bend.

## Case study

In this section, we consider the example of the longitudinal low-frequency axisymmetric L(0,1) mode in a small-bore pipe with a bend. The test pipe is the same as that used in “Numerical validation for the bi-orthogonality relationship” section. Assume that the L(0,1) mode with an excitation frequency of 30 kHz is excited in the straight part and then passes through the pipe bend. The mode structures in both the straight pipe and the pipe bend are calculated by using the SAFE method introduced in “Numerical validation for the bi-orthogonality relationship” section.

**Figure 5:**
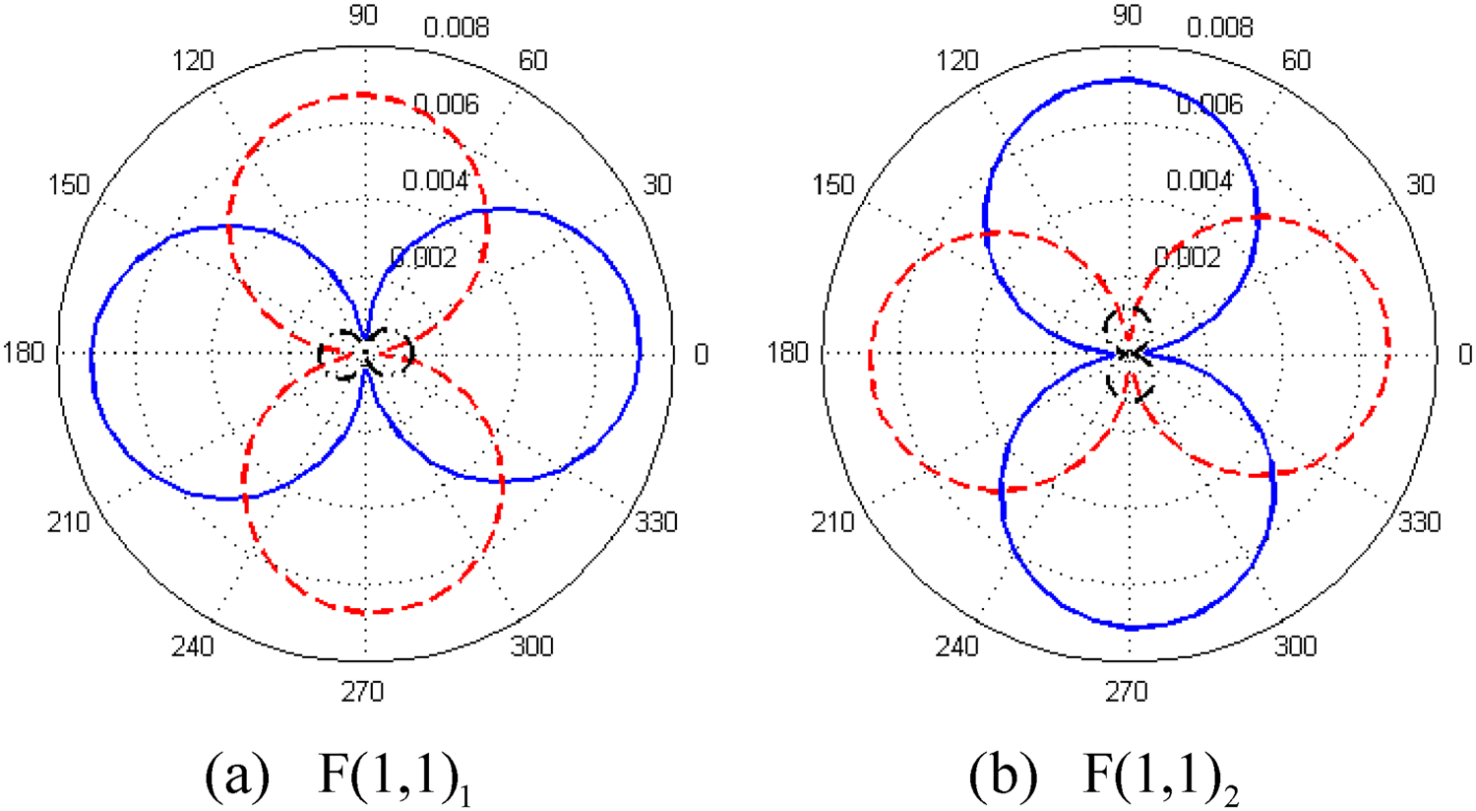
Displacement distributions of (**a**) F(1,1)_1_ and (**b**) F(1,1)_2_ along circumferential direction (the blue solid, red dotted, and black dashed-dotted lines show the displacements in the radial, circumferential, and axial directions, respectively).

The scattering at the $z^{\prime}_{1}$ interface is investigated first. The incident L(0,1) mode and all possible reflecting modes are expanded with the normalized modes in the pipe bend according to the bi-orthogonality relationships [Eq. (14)], and then the transfer matrix ${\mathbf{G}}_{lm}$ is constituted. Theoretically, the non-propagating reflecting and transmitting modes should be included in the calculation of ${\mathbf{G}}_{lm}$. However, because only the propagating modes are of concern in the practical testing scenario, we simplify the calculation of ${\mathbf{G}}_{lm}$ by ignoring the non-propagating modes. The input modes,are the incident L(0,1) and reflecting L(0,1), F(2,1)_1_, F(2,1)_2_, F(1,1)_1_, and F(1,1)_2_ modes. The output transmitting modes are the L_C_(0,1), F_C_(2,1)_1_, F_C_(2,1)_2_, F_C_(1,1)_1_, and F_C_(1,1)_2_ modes. F(1,1)_1_ and F(1,1)_2_ are the same modes because they have the same wavenumber. The difference between them is their circumferential orientations of displacement fields, as shown in Fig. 5; this is the same for F(2,1)_1_ and F(2,1)_2_.

Solving the eigenproblem of ${\mathbf{G}}_{lm} {\mathbf{G}}_{lm} ^{\prime}$ gives the reflection and transmission coefficients:

$$\begin{aligned} & {\mathbf{A}}_{l} = \left( {\begin{array}{*{20}c} {9936} & {572 - 718i} & { - 113 + 1i} & 5 & {37 + 3i} & {640 + 48i} \\ \end{array} } \right) \times 10^{ - 4} , \\ & {\mathbf{A}}_{m} = \left( {\begin{array}{*{20}c} { - {8137 + 5719}i} & {{30 + 234}i} & 0 & 0 & { - {515 + 2415}i} \\ \end{array} } \right) \times 10^{ - 4} . \\ \end{aligned}$$

${\mathbf{A}}_{l}$ is an eigenvector of ${\mathbf{G}}_{lm} {\mathbf{G}}_{lm} ^{\prime}$ corresponding to the eigenvalue of 0.2892 − 0.9633*i*, which should be one in theory.

Taking the absolute values of ${\mathbf{A}}_{l}$ and ${\mathbf{A}}_{m}$ gives

$$\begin{aligned} & \left| {{\mathbf{A}}_{l} } \right| = \left( {\begin{array}{*{20}c} {9936} & {918} & {113} & 5 & {38} & {642} \\ \end{array} } \right) \times 10^{ - 4} , \\ & \left| {{\mathbf{A}}_{m} } \right| = \left( {\begin{array}{*{20}c} {9946} & {{236}} & 0 & 0 & {{2469}} \\ \end{array} } \right) \times 10^{ - 4} . \\ \end{aligned}$$

From the reflection and transmission coefficients of the $z^{\prime}_{1}$ interface, we conclude the following: (i) ~ 10% of the incident L(0,1) mode is reflected, while other reflections are rather small; (ii) most of the incident L(0,1) mode is converted into the L_C_(0,1) mode, part is converted into the F_C_(1,1)_2_ mode, and all other mode conversions are negligible.

For the $z^{\prime}_{2}$ interface, there are three incident modes. For simplification, the modes with small amplitudes are ignored, and thus only the dominant L_C_(0,1) and F_C_(1,1)_2_ modes are considered here. Expanding the L_C_(0,1) and F_C_(1,1)_2_ modes with the normalized modes in straight section 3 give the transfer matrices ${\mathbf{G}}_{mn,1}$ and ${\mathbf{G}}_{mn,2}$. The input modes for L_C_(0,1) are the incident L_C_(0,1) and reflecting L_C_(0,1), F_C_(2,1)_1_, F_C_(2,1)_2_, F_C_(1,1)_1_, and F_C_(1,1)_2_ modes. The input modes for F_C_(1,1)_2_ are the incident F_C_(1,1)_2_ and reflecting L_C_(0,1), F_C_(2,1)_1_, F_C_(2,1)_2_, F_C_(1,1)_1_, and F_C_(1,1)_2_ modes. The output modes for both cases are the L(0,1), F(2,1)_1_, F(2,1)_2_, F(1,1)_1_, and F(1,1)_2_ modes.

Solving the eigenproblems of ${\mathbf{G}}_{mn,1} {\mathbf{G}}_{mn,1} ^{\prime}$ and ${\mathbf{G}}_{mn,2} {\mathbf{G}}_{mn,2} ^{\prime}$ gives the reflection and transmission coefficients for the L_C_(0,1) and F_C_(1,1)_2_ incidences:

$$\begin{aligned} & {\mathbf{A}}_{m,1} = \left( {\begin{array}{*{20}c} {9948} & { - 879 + 52i} & {77 - 82i} & 0 & 0 & {400 - 300i} \\ \end{array} } \right) \times 10^{ - 4} , \\ & {\mathbf{A}}_{n,1} = \left( {\begin{array}{*{20}c} { - {8240 + 5730}i} & {{18} - {65}i} & {3i} & {48 + 47i} & {{189 + 2646}i} \\ \end{array} } \right) \times 10^{4} , \\ & {\mathbf{A}}_{m,2} = \left( {\begin{array}{*{20}c} {9956} & {722 + 392i} & {145 - 409i} & 0 & 0 & {37 - 137i} \\ \end{array} } \right) \times 10^{ - 4} , \\ & {\mathbf{A}}_{n,2} = \left( {\begin{array}{*{20}c} { - {398 + 2399}i} & { - {118} - {597}i} & {9 + 24i} & { - 246 - 1i} & { - {7198} - {6418}i} \\ \end{array} } \right) \times 10^{ - 4} \\ \end{aligned}$$

which correspond to the eigenvalues of 0.2847 − 0.9351*i* and 0.0519 + 0.9121*i.*

Combining these scattering fields and the transmission coefficients of the $z^{\prime}_{1}$ interface gives the reflection and transmission coefficients of the $z^{\prime}_{2}$ interface:

$$\begin{aligned} {\mathbf{A}}_{m} & = \left( { - {0}{\text{.8137 + 0}}{.5719}i} \right) \cdot {\mathbf{A}}_{m,1} + \left( { - {0}{\text{.0515 + 0}}{.2415}i} \right) \cdot {\mathbf{A}}_{m,2} \\ & = \left( {\begin{array}{*{20}c} { - {8095 + 5689}i} & { - {513 + 2404}i} & {{554} - {391}i} & {{76 + 167}i} & 0 & 0 & { - {189 + 475}i} \\ \end{array} } \right) \times 10^{ - 4} , \\ \end{aligned}$$

$$\begin{aligned} {\mathbf{A}}_{n} & = \left( { - {0}{\text{.8137 + 0}}{.5719}i} \right) \cdot {\mathbf{A}}_{n,1} + \left( { - {0}{\text{.0515 + 0}}{.2415}i} \right) \cdot {\mathbf{A}}_{n,2} \\ & = \left( {\begin{array}{*{20}c} { - {2869} - {9595}i} & {{173 + 65}i} & { - {8} - {2}i} & {{53} - {70}i} & {{254 + 3453}i} \\ \end{array} } \right) \times 10^{ - 4} . \\ \end{aligned}$$

Thus, ${\mathbf{A}}_{l}$ and ${\mathbf{A}}_{n}$ give the reflection coefficients (${\mathbf{A}}_{r}$) and transmission coefficients (${\mathbf{A}}_{t}$) of the pipe bend at a frequency of 30 kHz. Taking the absolute values of ${\mathbf{A}}_{r}$ and ${\mathbf{A}}_{t}$ gives

$$\begin{aligned} & \left| {{\mathbf{A}}_{r} } \right| = \left( {\begin{array}{*{20}c} {0.0918} & {0.0113} & {0.0005} & {0.0038} & {0.0642} \\ \end{array} } \right), \\ & \left| {{\mathbf{A}}_{t} } \right| = \left( {\begin{array}{*{20}c} {1.0014} & {{0}{\text{.0185}}} & {0.0008} & {0.0088} & {{0}{\text{.3462}}} \\ \end{array} } \right), \\ \end{aligned}$$

where the reflection coefficients correspond to the reflecting L(0,1), F(2,1)_1_, F(2,1)_2_, F(1,1)_1_, and F(1,1)_2_ modes, and the transmission coefficients correspond to the transmitting L(0,1), F(2,1)_1_, F(2,1)_2_, F(1,1)_1_, and F(1,1)_2_ modes.

The reflection and transmission coefficients show that for unit normalized L(0,1) incidence, ~ 10% of the L(0,1) mode is reflected and more than 100% of it is transmitted, which means that the law of energy conservation is broken here. This happens because the principle of displacement and stress consistency at the interfaces does not hold in the NME framework.

However, although the scattering coefficients are not exact, inherent connections between normal modes in straight pipes and pipe bends are unveiled, and the main mode conversions at pipe bends are predicted correctly. In this case, it can be concluded that most of the incident L(0,1) mode passes through the pipe bend, some of it is reflected, and some converted into the F_C_(1,1)_2_ mode.

**Figure 6:**
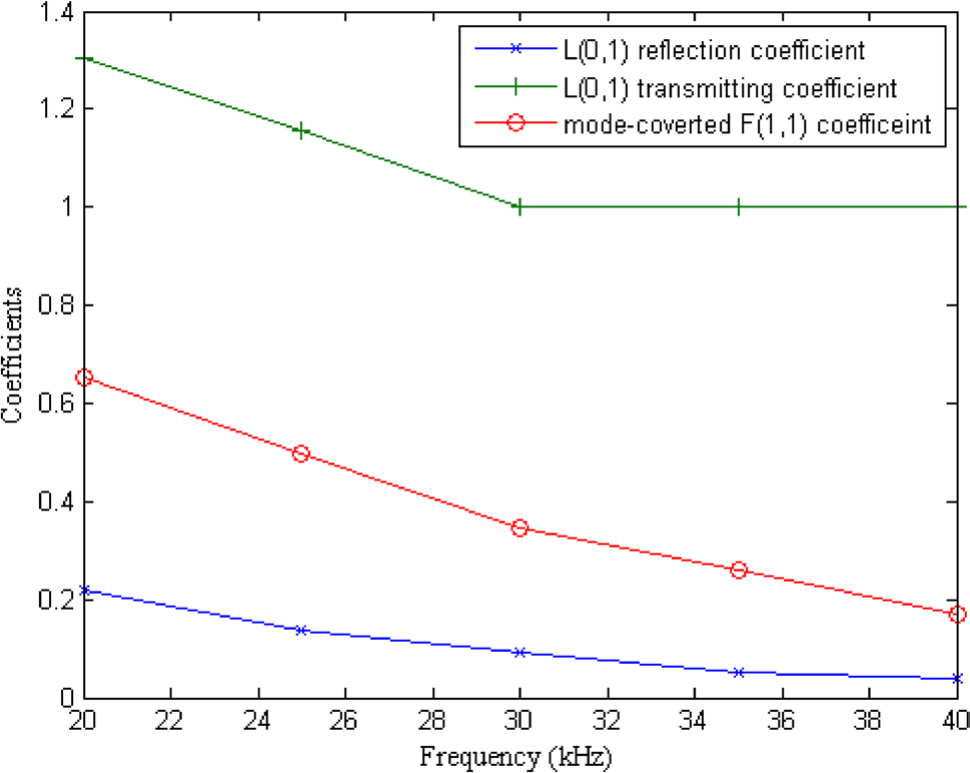
Evolution of the coefficients with respect to frequency.

The evolution of scattering coefficients of L(0,1) incidence with respect to frequency is shown in Fig. 6. As shown in Fig. 6, the L(0,1) bend reflection and the mode conversion from L(0,1) to F(1,1) increase significantly with the decrease of frequency, which is agrees with the experimental results reported in previous references. The L(0,1) transmitting coefficient is always bigger than 1, and approaches to 1 with the increase of frequency.

## Numerical simulations

**Figure 7:**
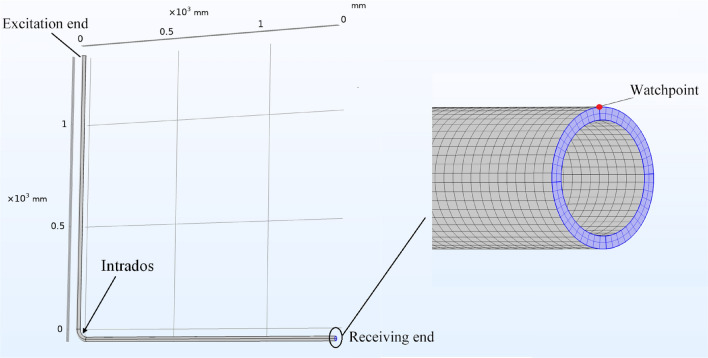
Finite-element modeling of test pipe.

To validate the results of the case study in “Case study” section, numerical simulations were conducted using the commercial finite-element analysis software COMSOL Multiphysics 5.6. The dimensions and material properties of the test pipe were those given in “Numerical validation for the bi-orthogonality relationship” section. The pipe was bent at its middle by an angle of 90°. Figure 7 shows the finite-element modeling of the pipe, which was meshed with two elements in the radial direction and 48 elements in the circumferential direction. The axial mesh spacing was set as 2 mm, which was chosen according to the mesh criterion of more than 20 nodes for the shortest wavelength of interest. The time step was set as 1 µs according to the criterion of $\Delta t < 1/\left( {20f_{\max } } \right)$, where *f*_*max*_ is the maximum frequency within a half-power bandwidth.

**Figure 8:**
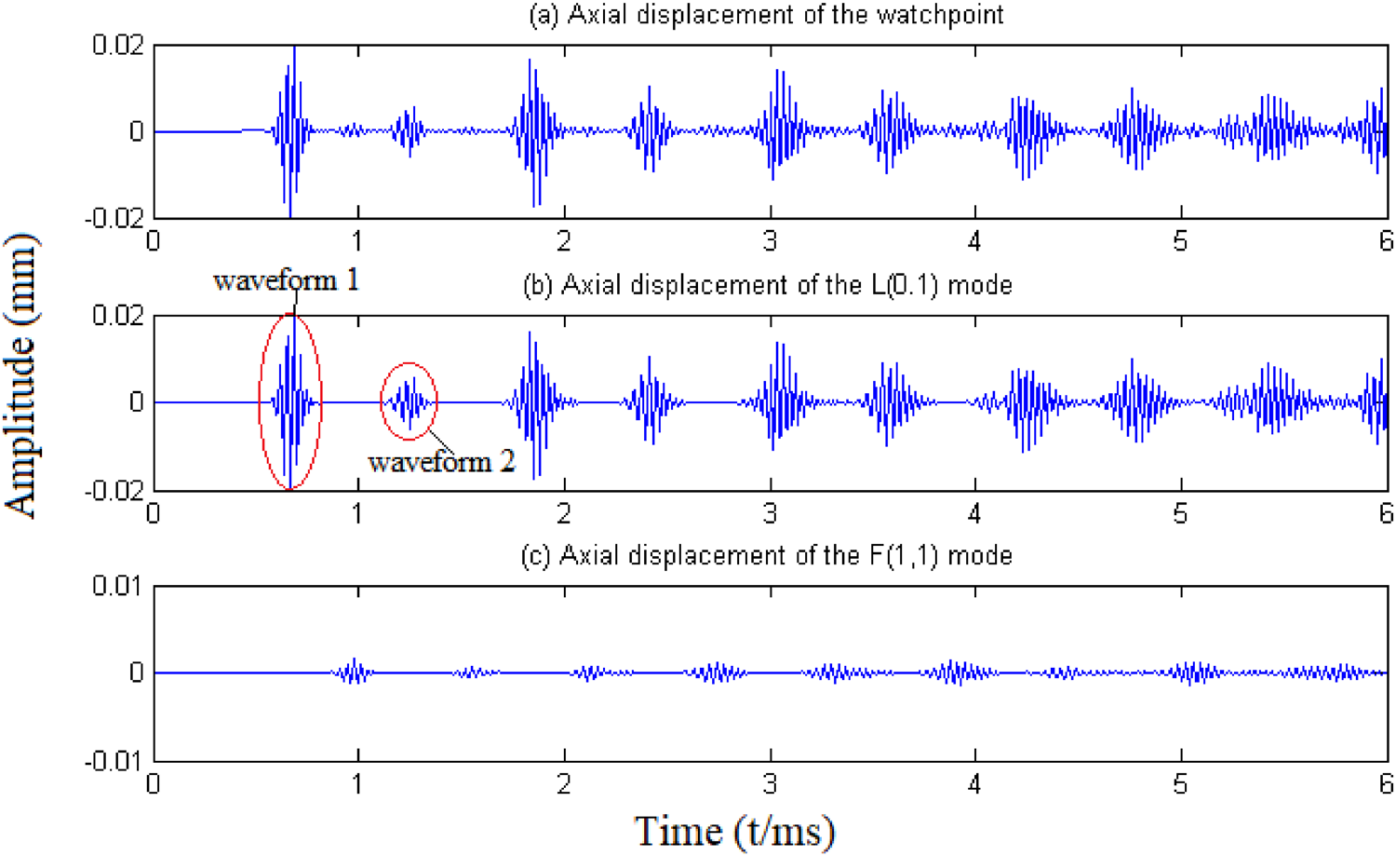
Time traces of axial displacement recorded at watchpoint.

A five-cycle sinusoidal tone burst modulated with the Hann window function at the excitation frequency of 30 kHz was applied on the cross section of one end of the pipe in the axial direction. The watchpoints were set on the other end of the pipe, as shown in Fig. 7. Figure 8 shows the time traces of the axial displacement recorded at the watchpoint which locates align to the intrados of the elbow (see Fig. 7): (a) is the complete time trace; (b) is the time trace of axisymmetric modes (the L(0,1) mode in this case) obtained by averaging the displacement of all nodes of the outer surface on the cross section; (c) is the time trace of the F(1,1) mode derived by subtracting the displacement of the watchpoint from that of its symmetrical counterpart.

Figure 8 shows that the recorded signal is mainly decomposed into waveforms of the L(0,1) and F(1,1) modes, indicating that no other remarkable mode conversions occur. Significant L(0,1) bend reflections are observed in Fig. 8b. The amplitude ratio of the first bend reflection [waveform 1 in Fig. 8b] to the first end reflection [waveform 2 in Fig. 8b] is ~ 0.2. In fact, the first bend reflection is composed of two L(0,1) bend reflections with different propagating routes but with the same flight time: one propagating from the excitation end to the bend, being reflected back to the excitation end, and then propagating through the bend to the receiving end; the other first propagating through the bend to the receiving end, turning back at the end, and then being reflected by the pipe bend. Therefore, ~ 10% of the incident L(0,1) mode is reflected by the bend. The converted F(1,1) mode seems rather small compared to the L(0,1) bend reflections, which is contrary to the theoretical prediction that a significant part of the L(0,1) mode is converted into the F(1,1) mode. This is because the F(1,1) mode has dominant displacements in the radial and circumferential directions but has a much smaller axial displacement (see Fig. 5).

In summary, the numerical simulation results agree well with the theoretical predictions. Although the theoretically derived scattering coefficients are not exact, the dominant mode conversions are obtained correctly.

## Experimental validation

**Figure 9:**
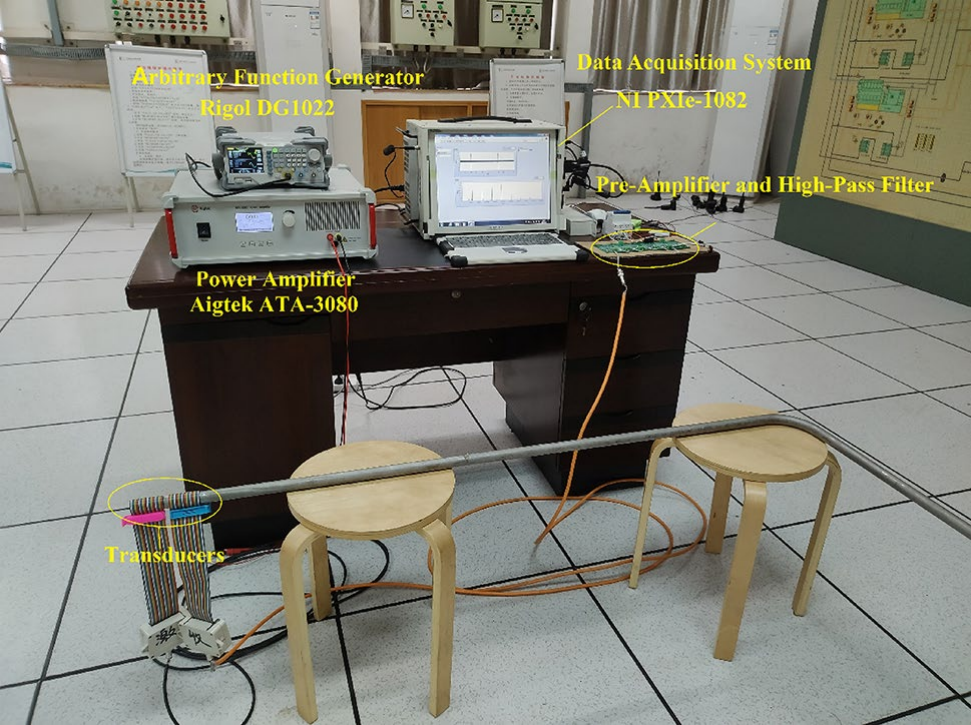
Experimental rig.

In this section, the scattering of the L(0,1) mode travelling through a bend is studied experimentally. The experimental rig is shown in Fig. 9. The test pipe was the same as that used in “Numerical validation for the bi-orthogonality relationship” section; this stainless-steel pipe was bent at its middle by an angle of 90° by using hot bending. A five-cycle 30-kHz tone burst was generated by an arbitrary function generator (Rigol DG1022) and subsequently magnified by a high-voltage power amplifier (Aigtek ATA-3080). The amplified signal was then sent to the transmitting transducer to excite the longitudinal guided waves in the pipe. The weak guided-wave signals were sensed by the receiving transducer and were pre-amplified and high-pass filtered before being acquired by the data acquisition system (NI PXIe-1082).

The transmitting and receiving transducers were placed on the same end of the pipe. Magnetostrictive patch transducers were employed. Four pre-magnetized iron-cobalt alloy strips of 70 mm length, 5 mm width, and 0.15 mm thickness were spaced equally around the circumference and bonded longitudinally on the pipe with epoxy glue. A 40-finger solenoid coil was wound over the patches to transmit and receive the signals.

**Figure 10:**
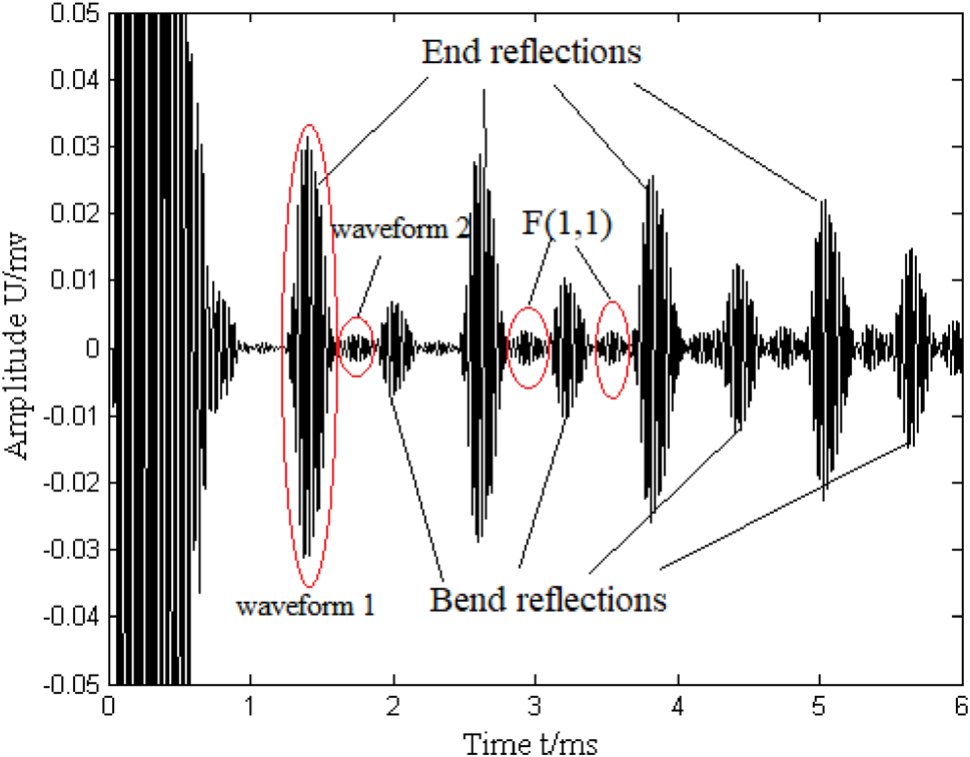
Guided-wave inspection of pipe with bend.

Figure 10 shows the experimental results. L(0,1) bend reflections are evident in the middle between two successive end reflections, this being because the bend was located at the middle of the pipe. Mode-converted F(1,1) is also observed, which can be confirmed simply by its flight time. The time difference between the L(0,1) end reflection (waveform 1 in Fig. 10) and its successive F(1,1) waveform (waveform 2 in Fig. 10) is ~ 0.33 ms. For one round trip, the incident L(0,1) travels through the bend twice (forth and back), and hence the mode conversion from L(0,1) to F(1,1) occurs twice. Waveform 2 is the scattered F(1,1) mode when the L(0,1) mode propagates back. According to the dispersion curves (see Fig. 2), the theoretical time difference between waveforms 1 and 2 is 0.3 ms, which agrees well with the experimental result.

The amplitude ratio of the first L(0,1) bend reflection in Fig. 10 to the first L(0,1) end reflection is ~ 0.2. In this pulse-echo experimental configuration, the first bend reflection in Fig. 10 is actually the second bend reflection, because the first bend reflection is masked by the initial pulse and cannot be distinguished. Thus, this first L(0,1) bend reflection is also composed of two L(0,1) bend reflections. Therefore, ~ 10% of the incident L(0,1) mode is reflected by the bend.

In summary, the experimental result that remarkable reflecting L(0,1) and mode-converted F(1,1) modes are scattered at the bend and ~ 10% of the incident L(0,1) mode is reflected by the bend agrees well with the numerical simulations, thereby validating the theoretical predictions.

## Conclusions

Herein, the scattering of guided waves propagating across pipe bends was studied. First, the bi-orthogonality relationship of normal modes in pipe bends was derived. Then, based on that relationship and considering that the displacement and stress fields at the interfaces between the straight and curved parts of a pipe should be consistent, the scattering problem was regarded as an eigenproblem of a transfer matrix. By solving this eigenproblem, the mode conversions at the interfaces were deduced. Combining the mode conversions at two interfaces of one bend gave the reflection and transmission coefficients of guided waves traveling through the bend. A case study of a low-frequency longitudinal guided wave (the L(0,1) mode) propagating across a pipe bend was given. Numerical simulations and experiments were further conducted to validate the theoretical predictions.

Because the normal modes are essentially the solutions to the governing equation of wave motions in waveguides, the normal modes cannot satisfy two different governing equations for different waveguides simultaneously, indicating that the principle of consistent displacement and stress fields does not hold in the NME framework. For the case of L(0,1) mode incidence, the theoretical prediction that ~ 10% of the incident mode is reflected, more than 100% is transmitted, and a remarkable part is converted into the F(1,1) mode is obviously contrary to the law of energy conservation. However, the NME-based derivation still reveals the inherent connections between normal modes in straight pipes and pipe bends, and it gives valuable information about the mode conversions at pipe bends. It is proved by numerical simulations and experiments that L(0,1) reflection and L(0,1)–F(1,1) conversion are the dominant mode conversions in this case.

## Data Availability

The datasets used and/or analysed during the current study available from the corresponding author on reasonable request.
